# Characteristics of Interval Colorectal Cancer: A Canadian Retrospective Population-Level Analysis from Newfoundland and Labrador

**DOI:** 10.3390/curroncol29120716

**Published:** 2022-11-24

**Authors:** Jessica J. Shanahan, Danielle M. LeBlanc, Emily R. Courage, Matthew G. K. Benesch, Kala E. Hickey, Katia A. Hartwig, Casey D. Armstrong, Reniel Engelbrecht, Mitchell G. Fagan, Mark R. Borgaonkar, David E. Pace

**Affiliations:** 1 Discipline of Surgery, Faculty of Medicine, Memorial University of Newfoundland, St. John’s, NL A1B 3V6, Canada; 2 Department of Surgical Oncology, Roswell Park Comprehensive Cancer Center, Buffalo, NY 14263, USA; 3 Discipline of Medicine, Faculty of Medicine, Memorial University of Newfoundland, St. John’s, NL A1B 3V6, Canada

**Keywords:** endoscopy, cancer screening, cancer surveillance, synoptic reporting

## Abstract

Interval colorectal cancers (I-CRCs) arise during the interval time period between scheduled colonoscopies. Predicting which patients are at risk of I-CRCs remains an elusive undertaking, but evidence would suggest that most I-CRCs arise from lesions missed on index endoscopy. The procedural factors that lead to missed lesions are numerous and lack consensus in the literature. In Canada, the province of Newfoundland and Labrador has the highest incidence of CRCs. In this study our aim was to examine I-CRCs (3–60 months after last colonoscopy) in NL through a population-level analysis covering 67% of the province from 2001–2018. We estimated the I-CRC rate to be up to 9.3%. Median age of I-CRC diagnosis was 67.1 years with an interval time of 2.9 years. About 57% of these tumors occurred proximal to the splenic flexure, with 53% presenting as local disease. No temporal differences were observed in interval time or tumor distribution. On univariate and multivariable logistical regression, risk of right-sided I-CRC did not correlate to the index colonoscopy indication, bowel preparation quality, size of largest polyp removed, colonoscopy completion rate, or stage at presentation. Improvements in synoptic reporting utilization and national registries are needed to identity risk factors and reduce I-CRC frequency.

## 1. Introduction

Colorectal cancer (CRC) is the third most common cancer, and second leading cause of cancer death worldwide [1]. Fortunately, the incidence of CRC has been decreasing over the last thirty years, attributable in part to screening and surveillance modalities, including colonoscopies [2]. The recent randomized control NordICC trial demonstrated that screening colonoscopies decreased the risk of CRC at 10 years by 18% [3]. Guidelines for CRC screening in average risk patients vary by country, but in general, endoscopic screening is part of the algorithm. In the United States, asymptomatic patients at average risk are recommended to undergo a colonoscopy every 10 years from 45 to 75 years of age [4], and in Canada, screening between the ages of 50–74 years old with a fecal occult blood test (FOBT) (either a guaiac FOBT or fecal immunochemical test (FIT)) every two years or flexible sigmoidoscopy every 10 years, with referral to colonoscopy in the event of an abnormal test [5]. Patients that are at higher-than-average risk, secondary to personal or family history of CRCs, personal history of polyps, history of inflammatory bowel disease, or genetic CRC predispositions undergo more frequent colonoscopy screening or surveillance, usually within defined 1–5 year interval criteria to detect polyps and other premalignant lesions prior to transformation [6].

Despite best screening efforts, interval CRCs (I-CRCs) account for approximately 5–6% of overall CRC incidence [7,8]. An I-CRC is defined by the World Endoscopy Organization as a “colorectal cancer diagnosed after a screening or surveillance exam in which no cancer is detected, and before the date of the next recommended exam” [9]. Compared to CRCs in general, studies on I-CRC outcomes are across the spectrum, ranging from better to worse outcomes [10]. Studies into the causes behind I-CRCs are limited and discordant, but they essentially fall into two broad categories. The first is procedural where lesions were inadvertently missed or inadequately resected, accounting for about 85% of I-CRCs by some estimates [11,12]. The second reason is biological, in that up to 20% of these cancers might have unpredictable behavior and arise within a time interval that precludes an effective interval screening [10]. Studies on the molecular features of these cancers are just beginning to emerge, but they have not demonstrated significant differences to control cancers with respect to microsatellite instability or differences on whole exome sequencing [8,10,13,14].

Newfoundland and Labrador (NL) is Canada’s most eastern province with a population of about 520,000, and it has the highest incidence and mortality rate for CRC in the country [15]. The reasons for this high incidence of CRC are like multifactorial, and research has shown this province to have among the highest rates of tobacco smoking [16], diabetes [17], pro-inflammatory diet [18], and germline genetic mutations [19,20] in Canada. Hence, in this study we aimed to estimate the I-CRC rate in NL, and compare the features of these I-CRCs to other Canadian and worldwide studies.

## 2. Materials and Methods

### 2.1. Study Design

Our study was initially designed to be a pan-NL retrospective populational-level analysis, and it received ethics approval from the Memorial University of Newfoundland Health Research Ethics Board (HREB #2016.165) prior to study commencement. Additional approval was required from each of the four health authorities in NL prior to the release of information. However, only two of these health authorities had the infrastructure to approve our proposals to access endoscopic and pathological records, limiting our study to patients residing in Labrador/Grenfell Health (encompassing Labrador and northern Newfoundland, 36,072 people per the 2016 Canadian Census), and Eastern Health (encompassing eastern Newfoundland, 313,267 people), representing approximately 67% of the total population (519,716 people). Because data were collected from patient charts for secondary use by an approved protocol, individual patient consent requirements were waived.

**Figure 1:**
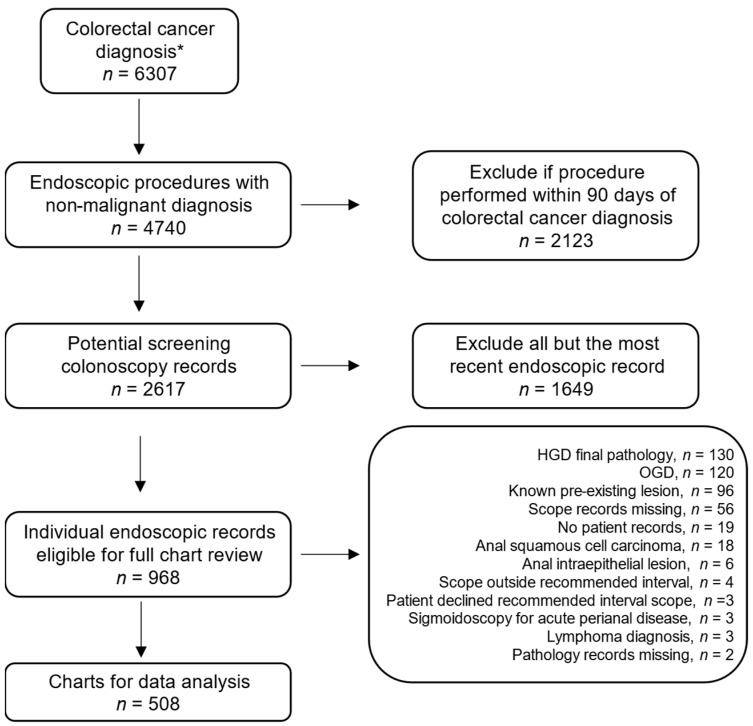
Flow chart of this study design with exclusion criteria. * Health charts numbers from 6307 patients with a colorectal cancer diagnosis where then searched for endoscopic procedures, which returned 4740 endoscopic records for further investigation. HGD, high grade dysplasia; OGD, oesophagogastroduodenoscopy (upper endoscopy).

Our study employed the assistance of the Newfoundland & Labrador Centre for Health Information (NLCHI, www.nlchi.nl.ca), and our study flowchart is depicted in Figure 1. Our time period covered any endoscopic procedures from 1 January 1996 to 31 March 2018. However, because endoscopic records were only electronically abstractable from 1 April 2001, our study period for cancer diagnosis was defined from 1 April 2001 to 31 March 2018. We then identified any colorectal cancer from the Newfoundland and Labrador Provincial Colorectal Cancer Database in this time period. Cancers were defined as being either in situ or malignant disease in disease sites identified by International Classification of Diseases for Oncology (ICD-O) topography codes (C18.0, cecum; C18.2 colon, ascending; C18.3 colon, hepatic flexure; C18.4, colon, transverse; C18.5, colon, splenic flexure; C18.6, colon, descending; C18.7, colon, sigmoid; C18.8, colon, overlapping region; C18.9, colon, not otherwise specified (NOS); C19.9, rectosigmoid junction; C20.9, rectum, NOS; C21.0, anus, NOS; C21.1, anal canal, C21.2, anus, cloacogenic zone; C21.8, rectum, anus and anal canal, overlapping lesion). This search retrieved 10,411 records for 9771 unique individuals in all of NL, of which 6420 cases in 6307 unique individuals were from the two health regions included in our analysis.

Our cohort was then designed from the health care numbers (HCN) of these cancer records by searching them against the NL Provincial Discharge Abstract Data (PDAD) for any endoscopic exam with a non-malignant most responsible diagnosis (MRD), in the proceeding 5 years prior to CRC diagnosis date. This was determined by eliminating any scope records with a ICD10-CA (Canada) MRD ranging from C00.00-C96.Z (malignant neoplasms). This search returned 4740 endoscopic procedures. Most I-CRC studies define an interval time period of at least 6 months (180 days) to distinguish between screening and diagnostic colonoscopies [13,21]. We chose to pursue a more generous interval time and excluded any procedures completed within 90 days prior to CRC diagnosis, leaving 2617 records. After excluding all but the most recent endoscopy record for patients with multiple records, 968 patient charts remained for full review using the NL electronic medical records system Meditech version 8 (Medical Information Technology, Inc., Canton, CA, USA). A complete list of chart review variables is presented in Supplemental Table S1.

### 2.2. Statistical Analysis

All statistical calculations were performed using Stata 15.1 (StataCorp LLC, College Station, TX, USA). For patient characteristics a Mann–Whitney U-test was used to compare two independent variables with a non-normal distribution, and a χ^2^ test and Fisher’s exact test for nominal variables, as appropriate. All odds ratios were calculated with 95% confidence intervals using univariate and multivariate logistic regression. All *p*-values were two sided, and the threshold of less than 0.05 was used to determine statistical significance.

## 3. Results

After a full review of endoscopic and pathologic records for the selected 968 patients, 508 patients were eligible for subsequent analysis (Figure 1). From a patient population of 6307 cancer diagnoses, our estimated I-CRC rate ranges from 8.1% to 9.3%, with the upper bound including all charts for which no patient records were missing (*n* = 585).

We then analyzed the demographic, endoscopic, and pathological results of the chart review (Table 1). Gender distribution was equal, with a median age of colonoscopy at 67.1 years, ranging from 34.2–86.3 years, with an interquartile range of 59.1–75.1 years. The median interval time from index colonoscopy to I-CRC diagnosis was 2.9 years, with a median age of diagnosis of 69.6 years. The primary indications for colonoscopy were rectal/acute bleeding, history of polyps, family history of CRC/polyps, and anemia or positive FOBT/FIT test, accounting for 63% of all scopes. Over 56% of these scopes were performed by general surgeons, reflective of the distribution of the endoscopists in this study. Colonoscopy completion to the cecum occurred 66.9% of the time, with a retroflexion in the rectum occurring 14.4% of the time. Withdrawal time was documented in 10.4% of cases. Bowel preparation regime was recorded for one-third of cases, and bowel preparation quality was not recorded 65.3% of the time. Excluding polyps, colonoscopies returned a normal finding 50.8% of the time. 50.2% of scopes identified at least one polyp. Polyp size was recorded for only one-quarter of cases. Sessile morphology was the most commonly identified at 13.2%, with adenomas being the most common histology. The largest polyp identified was proximal to the splenic flexure in 25.8% of cases, and distal to it 42.1% of the time. Over 95% of all CRCs were adenocarcinomas, with 24.8% occurring in the cecum, and 57% occurring proximal to the splenic flexure. Just over 50% of these cases presented as local disease (stage 2 or less) (Table 1).

We then repeated our analysis dichotomized by year index colonoscopy performed into groups 2001–2009 and 2010–2018 (Table 2), as synoptic reporting was introduced to Eastern Health in 2010 and about 90% of the endoscopic exams in this study occurred in this region. With the introduction of synoptic reporting, withdrawal time reporting increased from 9.7% to 22.3% of cases, and bowel prep regimen recording increased from 22.2% to 52.1%. Both the number of scopes with polyps identified (44.1% to 60.6%) and number of polyps per scope identified increased. There was no change in largest polyp size, however sessile polyp fraction increased from 10.6 to 17.6%. There was no change in the polyp location distribution. Further, there were no changes in any patient demographic or cancer location or stage between the two time periods (Table 2).

For patients where tumor location was known, we next investigated whether the odds of a right sided I-CRC versus a left sided I-CRC was associated with indication for index colonoscopy, poor bowel preparation, polyp size, incomplete scope, or stage at presentation. On both univariate and multivariable analyses, the odds ratio (OR) for any of these situations contributing to a right sided I-CRC over a left sided I-CRC was not statistically significant (Table 3).

We the repeated our analysis based on scope indication (Table 4). On both univariate and multivariable analyses, undergoing a colonoscopy for bleeding or anemia (including positive FOBT/FIT tests) did not predict risk of poor bowel preparation, large polyp, right-sided I-CRC, or advanced stage. When repeated based on a colonoscopy for personal history of polyps or CRC, the risk of an advanced tumor is significantly decreased with an OR of 0.60 (95% confidence interval 0.38–0.94), and is maintained on multivariable analysis (Table 4).

## 4. Discussion

The aim of our study was to provide the first examination of the characteristics of I-CRC in Newfoundland and Labrador, the province with the highest CRC rates in Canada. I-CRCs continue to represent a challenge to the management of CRC risk. Ideally, as the gold standard test, colonoscopy at patient individualized intervals ought to drop the I-CRC rate close to zero. This, however, is not the case. The exact factors contributing to I-CRCs are believed to be procedurally related, yet consensus in the literature is lacking.

We estimate our I-CRC rate to be between 8.1% to 9.3%. There have been three other Canadian studies, two in Ontario and one in Manitoba, calculating I-CRCs. Bressler et al. analyzed data from the Canadian Institute for Health Information, Ontario Health Insurance Program, and Ontario Cancer Registry from 1997–2002 and calculated an I-CRC rate from 2–6%, with rates highest for right-sided cancers [22]. This study was then repeated using data from 2000–2005, and recalculated the I-CRC rate at 9.0% [23]. The study from Manitoba calculated an I-CRC rate of 7.9%, ranging from 4.5% in the rectum/rectosigmoid to 14.4% in the cecum [24]. In all three of these studies, the time interval for a CRC was capped at 36 months and started either at 3 or 6 months from last endoscopy. Our study was designed to have a more generous interval from 3–60 months, recognizing that patients in most CRC screening algorithms will have a repeat colonoscopy booked at most in 5 years’ time. A 60-month endpoint is also employed in several large international I-CRC studies [25,26,27]. While our overall rate of up to 9.3% is consistent with the Canadian literature, the I-CRC rate is higher than reported in the worldwide literature closer to 5% [7,8,25]. In a meta-analysis of studies published after 2005, the I-CRC rate decreased nonsignificantly to about 3.7% [28].

Our study showed that 57% of I-CRCs occur proximal to the splenic flexure, and 47.6% proximal to the hepatic flexure, with a median age of 67.1 years. A large meta-analysis of twelve studies reporting 7912 I-CRCs calculated that these cancers were 2.4 times more likely to occur in the proximal colon, and were 15% more likely to the present over the age of 65 years [28]. This same analysis predicted that I-CRCs were less likely to present at an advanced stage with an OR 0.79 (95% CI 0.67–0.94) [28]. This agrees with our lack of correlation between any metrics examined and advanced tumor stage. Additional prior studies have not found a correlation between cecal intubation rates and I-CRC [29], also agreeing with our results (Table 3). Typically, up to 60% of I-CRCs are thought to arise from missed polyps [11]. Our results would also agree with this finding. In this study, we have used location of the largest polyp as a surrogate measure of polypectomy sites. Only 25.8% of the largest polyps identified arose in the right colon (proximal to splenic flexure), compared to 57% of I-CRC developing in the right colon (Table 2). Typically, only about 31% of all CRCs occur in the right colon, and this percentage has been stable over time [30].

Presumably, a high-quality colonoscopy by a skilled endoscopist should reduce the risk of I-CRC attributed to missed polyps. Missed polyps discovered on back-to-back colonoscopies is reported to be as high as 19% [31]. In this study, the OR of missing a polyp in the left colon was 0.62 compared to the right colon, but this did not reach statistical significance (95% CI 0.34–1.12, *p* = 0.11) [31]. A low adenoma detection rate (ADR) is a known predictor of I-CRC, with one report calculating an HR for an I-CRC for an ADR of less than 20% to be about 10-12 fold [29] relative to acceptable ADR rates of 30% in men and 20% in women [32]. The ADR in our centre was previously studied before and after implementation of the Canadian Association of Gastroenterology Skills Enhancement for Endoscopy (SEE) program in 2015, which resulted in a non-significant improvement in ADR from 31.8% to 35.3% for surgeons and no change for the gastroenterology group at 32.8% [33]. Therefore, despite having an excellent ADR and an increase in polyp yield increased over the time course of our study, there was no effect on the location distribution of I-CRCs.

Unfortunately, bowel preparation regimen and quality were poorly recorded in our study, precluding us from making conclusions on the correlation between preparation quality and I-CRCs. However, there are several investigations, including a randomized controlled trial, that failed to show statistically significant differences in ADRs between patients with fair bowel preparation and those with excellent preparation [34,35,36,37]. This is likely due to a combination of the endoscopist spending additional time examining the mucosal surface while washing the colon and/or re-booking the patient in is a short interval for a repeat exam with a better preparation.

This study does have several limitations. First, it is a retrospective analysis which is prone to selection bias. However, we have employed a population-level analysis approach with a generous time interval with no exclusion criteria to identify every possible case of I-CRC. The largest limitation to our data collection is missing information. All charts prior to 2010 in our study were digitized copies of paper charts, resulting in inconsistent documentation of most procedural parameters. With the introduction of synoptic reporting in the largest NL health region in 2010, recording of many of these parameters improved, such as documentation of withdrawal time and bowel preparation adequacy. However, documentation rates were still too low to enable meaningful interpretation. In a broader context, rigorous documentation of quality indicators and procedural outcomes and findings is critical to improving the quality of patient care [23]. The ability to recognize institutional and regional shortcomings requires robust data collection, which in turn would also help in researching relatively rare phenomenon like I-CRC. Systematic collection of data through synoptic reports can also build national-level registries to study regional and temporal trends. Thus far, colorectal registries in North America are fractionated, and lack the power of long-standing registries that exist in European countries [38].

## 5. Conclusions

In conclusion, this study has calculated the rate of I-CRCs and outlined patient demographic, endoscopic, and pathological characteristics in NL, which has the highest rate of CRCs in Canada. Further research into temporal and procedural patterns that might account and predict patients at risk for I-CRCs will require additional longitudinal data that is collected systematically and uniformly across different health authorities.

## Figures and Tables

**Table 1:** Patient, endoscopy, and pathological characteristics of analyzed charts.

N	508
**Sex (%)**	
Male	255 (50.2)
Female	253 (49.8)
**Age at colonoscopy (Years)**	
Mean (SD)	66.4 (11.5)
Median	67.1
Range (IQR)	34.2–86.3 (59.1–75.1)
**Interval time (Years)**	
Mean (SD)	2.9 (1.4)
Median	2.9
Range (IQR)	0.3–5.0 (1.4–4.0)
**Age at CRC diagnosis (Years)**	
Mean (SD)	69.1 (11.4)
Median	69.6
Range (IQR)	35.3–90.0 (61.7–77.8)
**Indication for scope (%)**	
Rectal/acute bleed	94 (18.5)
History of polyps	91 (17.9)
Family history CRC/polyps	73 (14.4)
Anemia/FOBT+/FIT+	63 (12.4)
Altered bowel habits	48 (9.5)
Abdominal pain	32 (6.3)
History/Suspected IBD (screening)	22 (4.3)
History of CRC	25 (4.9)
Abnormal colonic imaging	17 (3.4)
CRC genetic syndrome	15 (3.0)
Asymptomatic screening	11 (2.2)
Diverticular flare (follow-up)	4 (0.8)
Not stated	13 (2.6)
**Specialty (%)**	
Surgeon	286 (56.3)
Gastroenterologist	148 (29.1)
Internist	58 (11.4)
Not stated	16 (3.2)
**Trainee present (%)**	
Yes	15 (3.0)
No	48 (9.5)
Not stated	445 (87.5)
**Full colonoscopy completed (%)**	
Yes	340 (66.9)
No	163 (32.1)
Flexible sigmoidoscopy	70 (13.8)
Incomplete	93 (18.3)
Not stated	5 (1.0)
**Rectal retroflex performed (%)**	
Yes	73 (14.4)
No	435 (85.6)
**Withdrawal time recorded (%)**	
Yes	53 (10.4)
No	455 (89.6)
**Adverse effects (%)**	
Yes	17 (3.3)
No	264 (52.0)
Not stated	227 (44.7)
**Bowel prep regimen recorded (%)**	
Yes	174 (34.3)
No	334 (65.7)
**Bowel prep quality (%)**	
Excellent/very good	34 (6.7)
Good	73 (14.4)
Fair/poor	47 (9.2)
Very poor/bad	22 (4.3)
Not stated	332 (65.3)
**Antispasmodic used (%)**	
Yes	22 (4.3)
No	193 (38.0)
Not stated	293 (57.7)
**Non-polyp findings (%)**	
Normal	258 (50.8)
Benign (diverticulosis, angiodysplasia)	207 (40.7)
Inflammatory bowel disease	23 (4.5)
Bleeding	1 (0.2)
Not stated	19 (3.7)
**Polyps identified (Number identified) (%)**	
Yes	255 (50.2)
1–2	163 (32.1)
3–4	43 (8.5)
5–10	22 (4.3)
>10	27 (5.3)
No	234 (46.1)
Not stated	19 (3.7)
**Polyps removed or biopsied (%)**	
Yes	239 (47.0)
No	262 (51.6)
Not stated	7 (1.4)
**Largest polyp size (%)**	
1–5 mm	46 (9.1)
6–10 mm	36 (7.1)
11–20 mm	26 (5.1)
>20 mm	13 (2.6)
Not stated	387 (76.2)
**Polyp morphology (%)**	
No polyp	252 (49.6)
Sessile	67 (13.2)
Pedunculated	22 (4.3)
Flat	20 (3.9)
Not stated	147 (28.9)
**Polyp histology (%)**	
No polyp	252 (49.6)
Adenoma	161 (31.7)
Hyperplastic	49 (9.6)
Serrated	16 (3.2)
Inflammatory	5 (1.0)
Lymphoid	1 (0.2)
Normal mucosa	3 (0.6)
Not determined (polyp not retrieved)	21 (4.1)
**Polyp location (largest) (%)**	
Cecum	12 (4.7)
Right colon (not cecum)	41 (16.0)
Transverse colon	13 (5.1)
Left colon (not rectum)	80 (31.2)
Rectum	28 (10.9)
Pan colonic	46 (18.0)
Not stated	36 (14.1)
**Interval cancer pathology (%)**	
Adenocarcinoma	484 (95.3)
Other	19 (3.7)
Not stated	5 (1.0)
**Interval cancer location (%)**	
Cecum	126 (24.8)
Right colon (not cecum)	116 (22.8)
Transverse colon	50 (9.8)
Left colon (not rectum)	116 (22.8)
Rectum	78 (15.4)
Multiple cancers	7 (1.4)
Liver metastasis	1 (0.2)
Not stated	14 (2.8)
**Interval cancer stage (%)**	
Stage 0 (in situ)	39 (7.7)
Stage 1	108 (21.3)
Stage 2	119 (23.4)
Stage 3	124 (24.4)
Stage 4	77 (15.2)
Not stated	41 (8.1)

**Table 2:** Patient, endoscopy, and pathological characteristics of analyzed charts dichotomized by time period.

	2001–2009	2010–2018	*p*-Value
**N**	320	188	-
**Sex (%)**			
Male	153 (47.8)	102 (54.3)	0.16
Female	167 (52.2)	86 (45.7)	
**Age at colonoscopy (Years)**			
Mean (SD)	65.7 (12.2)	67.7 (10.2)	0.06
Median	65.8	68.5	
Range (IQR)	30.8–86.3 (57.8–75.0)	37.6–86.4 (60.5–74.4)	
**Interval time (Years)**			
Mean (SD)	2.7 (1.4)	2.7 (1.4)	0.6
Median	2.9	3	
Range (IQR)	0.3–5.0 (1.4–4.0)	0.2–4.9 (1.4–3.9)	
**Age at CRC diagnosis (Years)**			
Mean (SD)	68.4 (12.1)	70.3 (10.1)	0.06
Median	68.9	70.4	
Range (IQR)	34.0–89.6 (61.0–77.9)	40.9–90.5 (63.7–77.8)	
**Specialty (%)**			
Surgeon	99 (30.9)	49 (26.1)	0.5
Gastroenterologist	38 (11.9)	20 (10.6)	
Internist	172 (53.8)	114 (60.6)	
Not stated	11 (3.4)	5 (2.7)	
**Trainee present (%)**	8 (2.5)		
Yes	24 (7.5)	7 (3.7)	0.1
No	288 (90.0)	24 (12.8)	
Not stated		157 (83.5)	
**Full colonoscopy completed (%)**			
Yes	194 (60.6)	146 (77.7)	<0.001
No	123 (38.4)	40 (21.3)	
Flexible sigmoidoscopy	65 (20.3)	16 (8.5)	
Incomplete	58 (18.1)	24 (12.8)	
Not stated	3 (0.9)	2 (1.1)	
**Rectal retroflex performed (%)**			
Yes	31 (9.7)	42 (22.3)	<0.001
No	289 (90.3)	146 (77.7)	
**Withdrawal time recorded (%)**			
Yes	12 (3.8)	41 (21.8)	<0.001
No	308 (96.2)	147 (78.2)	
**Adverse effects (%)**			
Yes	14 (4.4)	3 (1.6)	<0.001
No	138 (43.1)	126 (67.0)	
Not stated	168 (52.5)	59 (31.4)	
**Bowel prep regimen recorded (%)**			
Yes	71 (22.2)	98 (52.1)	<0.001
No	249 (77.8)	90 (47.9)	
**Bowel prep quality (%)**			
Excellent/very good	9 (2.8)	25 (13.3)	<0.001
Good	25 (7.8)	48 (25.5)	
Fair/poor	30 (9.4)	17 (9.0)	
Very poor/bad	16 (5.0)	6 (3.2)	
Not stated	240 (75.0)	92 (48.9)	
**Antispasmodic used (%)**			
Yes	14 (4.4)	8 (4.3)	<0.001
No	90 (28.1)	103 (54.8)	
Not stated	216 (67.5)	77 (41.0)	
**Polyps identified** **(Number Identified) (%)**			
Yes	141 (44.1)	114 (60.6)	<0.001
1–2	90 (28.1)	73 (38.8)	0.002
3–4	19 (5.9)	24 (12.8)	
5–10	12 (3.8)	10 (5.3)	
>10	20 (6.2)	7 (3.7)	
No	163 (50.9)	71 (37.8)	
Not stated	16 (5.0)	3 (1.6)	
**Polyps removed or biopsied (%)**			
Yes	129 (40.3)	110 (58.5)	<0.001
No	186 (58.1)	76 (40.4)	
Not stated	5 (1.6)	2 (1.1)	
**Largest polyp size (%)**			
1–5 mm	20 (6.2)	26 (13.8)	0.05
6–10 mm	23 (7.2)	13 (6.9)	
11–20 mm	15 (4.7)	11 (5.9)	
>20 mm	7 (2.2)	6 (3.2)	
Not stated	255 (79.7)	132 (70.2)	
**Polyp morphology (%)**			
No polyp	178 (55.6)	74 (39.4)	<0.001
Sessile	34 (10.6)	33 (17.6)	
Pedunculated	16 (5.0)	6 (3.2)	
Flat	7 (2.2)	14 (7.4)	
Not stated	85 (26.6)	61 (32.4)	
**Polyp location (largest) (%)**			
Cecum	6 (4.2)	6 (5.3)	0.06
Right colon (not cecum)	19 (13.4)	22 (19.3)	
Transverse colon	8 (5.6)	5 (4.4)	
Left colon (not rectum)	52 (36.6)	28 (24.6)	
Rectum	13 (9.2)	15 (13.2)	
Pan colonic	19 (13.4)	27 (23.7)	
Not stated	25 (17.6)	11 (9.6)	
**Interval cancer location (%)**			
Cecum	85 (26.6)	41 (21.8)	0.85
Right colon (not cecum)	70 (21.9)	46 (24.5)	
Transverse colon	29 (9.1)	21 (11.2)	
Left colon (not rectum)	74 (23.1)	42 (22.3)	
Rectum	47 (14.7)	31 (16.5)	
Multiple cancers	4 (1.2)	3 (1.6)	
Liver metastasis	1 (0.3)	0 (0.0)	
Not stated	10 (3.1)	4 (2.1)	
**Interval cancer stage (%)**			
Stage 0 (in situ)	30 (9.4)	9 (4.8)	0.26
Stage 1	67 (20.9)	41 (21.8)	
Stage 2	71 (22.2)	48 (25.5)	
Stage 3	73 (22.8)	51 (27.1)	
Stage 4	54 (16.9)	23 (12.2)	
Not stated	25 (7.8)	16 (8.5)	

**Table 3:** Derived univariate and multivariable odds ratios for a right sided I-CRC.

	Univariate	*p*-Value	Multivariable	*p*-Value
**Scope reason**				
Polyp/CRC history	1.26 (0.77–2.07)	0.35	1.41 (0.83–2.41)	0.21
**Bowel preparation**				
Poor/bad	1.75 (0.94–3.29)	0.08	1.82 (0.74–4.44)	0.19
Not stated	1.16 (0.75–1.80)	0.5	1.15 (0.61–2.17)	0.66
**Largest polyp** **(>1 cm)**	0.76 (0.40–1.47)	0.42	0.71 (0.28–1.79)	0.47
**Complete scope (No)**	0.98 (0.69–1.43)	0.9	1.46 (0.83–2.28)	0.21
**Stage** **(Advanced/not stated)**	1.17 (0.82–1.67)	0.38	1.38 (0.84–2.28)	0.21

Reference categories: Scope reason (bleeding/anemia), Bowel prep (excellent/good/fair), Largest polyp (≤1 cm or not stated), Complete scope (Yes). Stage (Localized, stage 2 and under).

**Table 4:** Derived univariate and multivariable odds ratios for a colonoscopy by indication.

	Univariate	*p*-Value	Multivariable	*p*-Value	Univariate	*p*-Value	Multivariable	*p*-Value
**Scope indication**	Bleeding/anemia				Personal history polyp/CRC			
**Bowel preparation**								
Poor/bad	1.83 (0.92–3.61)	0.08	1.77 (0.87–3.57)	0.11	0.68 (0.34–1.38)	0.29	0.76 (0.36–1.60)	0.47
Not stated	1.83 (1.10–3.07)	0.02	1.77 (1.04–3.03)	0.04	0.65 (0.40–1.07)	0.09	0.72 (0.43–1.21)	0.54
**Largest polyp** **(>1 cm)**	1.28 (0.64–2.53)	0.48	1.38 (0.65–2.94)	0.4	0.86 (0.39–1.93)	0.72	0.83 (0.34–2.01)	0.65
**Complete scope (No)**	1.92 (1.29–2.85)	0.001	1.93 (1.28–2.93)	0.2	0.25 (0.14–0.44)	<0.001	0.29 (0.16–0.52)	<0.001
**Tumor location (Right)**	0.95 (0.64–1.40)	0.79	0.96 (0.64–1.44)	0.86	1.28 (0.83–1.99)	0.26	1.31 (0.83–2.06)	0.25
**Stage (Advanced/not stated)**	1.13 (0.77–1.64)	0.53	1.12 (0.75–1.66)	0.59	0.60 (0.39–0.92)	0.02	0.60 (0.38–0.94)	0.03

Reference categories: Bowel prep (excellent/good/fair), Largest polyp (≤1 cm or not stated), Complete scope (Yes), Tumor location (Left, distal to splenic flexure) Stage (Localized, stage 2 and under).

## Data Availability

Raw data would be available through application and review by Memorial University of Newfoundland Health Research Ethics Board.

## Supplementary Materials

The following supporting information can be downloaded at: https://www.mdpi.com/article/10.3390/curroncol29120716/s1, Table S1: List of variables analyzed in chart review.
