# Supplementary material for: Characteristics of Interval Colorectal Cancer: A Canadian Retrospective Population-Level Analysis from Newfoundland and Labrador
Source: Curr Oncol. 2022 Nov 24;29(12):9150–62. doi: 10.3390/curroncol29120716 (PMC9776431; doi:10.3390/curroncol29120716)
Supplement: Supplementary file 1 [file curroncol-29-00716-s001.zip › curroncol-2031323-supplementary.pdf]

Supplemental Table S1. List of variables analyzed in chart review.

Variable	Coding
**Demographic Variables**	
**Study ID number**	numerical
**Date of Birth (DOB)**	(YY-MM-DD)
**Date of Death (DOD)**	(YY-MM-DD)
**Gender**	1- Male; 2- Female
**Date of Procedure (DOP)**	(YY-MM-DD)
**Indication for Colonoscopy**	1- Family history of CRC; 2- Personal history of CRC; 3- Family history of polyps; 4- Personal history of polyps; 5- No family history of CRC/polyps cancer screening; 6- known/suspected IBD; 7- Altered bowel habits; 8- Abdominal pain; 9- Not stated 10- Anemia of Chronic disease/Anemia/FOB+; 11- unidentified mass; 12- f/u diverticular disease; 13- acute bleeding; 14- rectal bleeding; 15- IBD Cancer screening; 16- Abnormal colonic imaging; 17- known carrier or a genetic CRC syndrome
**Colonoscopy Variables**	
**Location**	Health regions (categorical)
**Did a trainee participate**	1- Yes; 2- No; 9- Not stated
**Endoscopist specialty**	1- Gastroenterologist; 2- Internist; 3- Surgeon; 4- Family doctor; 9- Not stated
**Was colonoscopy complete**	1- Yes; 2- No; 9- Not stated
**How was completeness documented**	1- Landmark description; 2- Intubation of ileum; 3- Photograph; 4- Biopsy of ileum; 5- Not complete; 9- Not documented
**If incomplete, what was the reason**	1- Redundancy; 2- Obstruction; 3- Patient discomfort; 4- Complication; 5- Poor preparation; 6- Diverticulosis; 7- Not applicable (was complete) 9- Not stated
**Maximal insertion**	1- Cecal intubation; 2- Incomplete; 3- Flexible sigmoidoscopy; 9- Not stated
**Documentation of rectal retroflexion**	1- Yes; 2- No
**Withdrawal time recorded**	1- Yes; 2- No
**Withdrawal time in minutes and seconds**	numerical
**Adverse events**	1- Yes; 2- No; 9- Not stated
**Bowel prep type**	1- Pico Salax; 2- Purgodan; 3- Peglyte; 4- Golytely; 5- Bipeglyte; 6- fleet enema 7- picosalax, bisacodyl/ducolax 8- bisacodyl/ducolax ; 9- Not stated

**Bowel prep quality**	1- Excellent/Very good; 2- Good; 3- Fair/Poor; 4- Very poor/Bad; 9- Not stated
**Findings (excluding polyps)**	1- Normal; 2- IBD (active or in remission); 3- other benign (diverticulosis, angiodysplasia); 4- Colonoscopy not performed/started; 5- Normal but incomplete; 6- Perforation; 7- bleeding 9- Not stated 10- none
**Sedation used**	1- Yes; 2- No; 9- Not stated
**Sedation agent**	1- Propofol; 2- Versed; 3- Fentanyl; 4- Valium; 5- Demerol; 6- Demerol, valium, 7- fentanyl, Versed; 8 Versed, Demerol; 9- other; 10- Not stated; 11- none
**Was antispasmodic used**	1- Yes; 2- N-o; 9 Not stated
**Polyp Variables at Index Colonoscopy**	
**Was a polyp identified**	1- Yes; 2- No; 3- biopsied; 9- Not stated
**Was a polyp removed**	1- Yes; 2- No; 9- Not stated
**Total number of polyps identified**	numerical, 99- multiple
**Total number of polyps removed**	numerical, 99-multiple
**Largest polyp size (mm)**	numerical (blank is not stated)
**Polyp morphology**	1- Pedunculated; 2- Sessile; 3- Flat; 4- Umbilicated/ulcerated; 5- No polyp; 9- Not stated
**Polyp histology**	1- No polyp; 2- Adenoma; 3- Hyperplastic; 4- Inflammatory; 5- Lymphoid; 6- Normal; 7- Not determined (polyp not retrieved); 8- Malignant; 9- Leiomyoma; 10- polyp not removed; 11- Serrated (SSA/P); 12- Serrated with dysplasia; 13- Traditional serrated adenoma (TSA); 14- ganglioneuroma
**Polyp location (largest polyp)**	1- Cecum; 2- Ascending; 3- Hepatic flexure; 4- Transverse; 5- Splenic flexure; 6- Descending colon; 7- Sigmoid; 8- Rectum; 9- Not stated; 10- Pan colon; 11- Right colon; 12- Left colon; 13- no polyps
**Polypectomy Technique**	1- Snare/excision with cautery; 2- Snare/excision without cautery; 3- Cold/excisional biopsy; 4- Snare and hot excisional biopsy 5- Hot biopsy/fulguration; 6- Endoscopic mucosal resection (if any injection of fluid to create a pseudostalk); 7- N/A (no polyp); 8- Excised/snared with no further description; 9- Polyp not removed
**Interval Colorectal Cancer Variables**	
**Pathology**	1- Adenocarcinoma; 2- Adenosquamous carcinoma; 3- Spindle cell carcinoma; 4- Squamous cell (epidermoid) carcinoma; 5- Undifferentiated carcinoma; 6- Other (neuroendocrine, lymphoma, mesenchymal, etc.); 8- N/A (No CRC); 9- Not stated
**Location**	1- Cecum; 2- Ascending; 3- Hepatic flexure; 4- Transverse; 5- Splenic flexure; 6- Descending colon; 7- Sigmoid; 8- Rectosigmoid; 9- Rectum; 10- N/A (No CRC); 11- Not stated; 12- Multiple cancers; 13- liver met
**Stage**	1- Stage 1; 2- Stage 2; 3- Stage 3; 4- Stage 4; 8; N/A (No CRC); 9- Not stated
